# Effects of Chronic Sleep Restriction on Transcriptional Sirtuin 1 Signaling Regulation in Male Mice White Adipose Tissue

**DOI:** 10.3390/cimb46030138

**Published:** 2024-03-07

**Authors:** Marco Rendine, Paolo Cocci, Luisa de Vivo, Michele Bellesi, Francesco Alessandro Palermo

**Affiliations:** 1 School of Biosciences and Veterinary Medicine, University of Camerino, 62032 Camerino, Italy; marco.rendine@unimi.it (M.R.); paolo.cocci@unicam.it (P.C.); michele.bellesi@unicam.it (M.B.); 2 Department of Food, Environmental and Nutritional Sciences (DeFENS), Università degli Studi di Milano, 20133 Milan, Italy; 3 School of Pharmacy, University of Camerino, 62032 Camerino, Italy; luisa.devivo@unicam.it; 4 School of Physiology, Pharmacology and Neuroscience, University of Bristol, Bristol BS8 1QU, UK

**Keywords:** sleep restriction, SIRT1 expression, lipid homeostasis

## Abstract

Chronic sleep restriction (CSR) is a prevalent issue in modern society that is associated with several pathological states, ranging from neuropsychiatric to metabolic diseases. Despite its known impact on metabolism, the specific effects of CSR on the molecular mechanisms involved in maintaining metabolic homeostasis at the level of white adipose tissue (WAT) remain poorly understood. Therefore, this study aimed to investigate the influence of CSR on sirtuin 1 (SIRT1) and the peroxisome proliferator-activated receptor γ (PPARγ) signaling pathway in the WAT of young male mice. Both genes interact with specific targets involved in multiple metabolic processes, including adipocyte differentiation, browning, and lipid metabolism. The quantitative PCR (qPCR) results demonstrated a significant upregulation of SIRT-1 and some of its target genes associated with the transcriptional regulation of lipid homeostasis (i.e., PPARα, PPARγ, PGC-1α, and SREBF) and adipose tissue development (i.e., leptin, adiponectin) in CSR mice. On the contrary, DNA-binding transcription factors (i.e., CEBP-β and C-myc), which play a pivotal function during the adipogenesis process, were found to be down-regulated. Our results also suggest that the induction of SIRT1-dependent molecular pathways prevents weight gain. Overall, these findings offer new, valuable insights into the molecular adaptations of WAT to CSR, in order to support increased energy demand due to sleep loss.

## 1. Introduction

White adipose tissue (WAT) is one of the most important organs involved in the regulation of metabolic homeostasis. WAT influences both endocrine homeostasis and immune system function by secreting several adipokines, such as leptin [1], adiponectin [2], TNFα [3], and resistin [4], among many others [5]. In addition, WAT adipocytes show significant phenotypic plasticity, as demonstrated by their involvement in the browning phenomenon (i.e., a switch from white to beige-like adipocytes) [6]. WAT transcriptional signaling is finely orchestrated by networks of transcription factors that regulate the expression of several target genes. In this regard, sirtuin 1 (SIRT1) is a NAD+-dependent deacetylase involved in regulating key metabolic pathways in adipocytes [7,8]. The SIRT1-related molecular network includes several fatty acid sensors, such as peroxisome proliferator-activated receptors (PPARs) [9], and transcription factor coregulators, such as peroxisome proliferator-activated receptor gamma coactivator 1-alpha (PGC1α) and a PR domain containing 16 (PRDM16) [7]. It has recently been reported that the interplay between PPARγ and SIRT1 seems to be critical for WAT browning [10]. This phenotypic change is likely to be protective against obesity-associated metabolic disorders [11]. The importance of SIRT1 signaling in WAT is supported by several in vitro and in vivo studies [10,12]. In fact, the deregulation of the SIRT1-mediated pathway is closely associated with a range of metabolic alterations, as observed in a SIRT1 adipose-specific knockdown mouse model which resulted in fat accumulation, alterations in glucose and lipid metabolism, as well as impaired insulin sensitivity, and the development of obesity [12]. There is also evidence that SIRT1 has an anti-inflammatory effect on WAT by inhibiting the expression of inflammatory response genes [13,14]. One of the environmental factors that can modulate metabolism and has been linked to increased risks of developing obesity is sleep deprivation. Many studies described an inverse or U-shaped relationship between sleep duration and weight gain [15,16,17], with sleep deprivation being associated with increased hunger and appetite, through affecting the leptin and ghrelin signaling pathways [18], an increased intake of caloric food [19,20], and a higher Body Mass Index (BMI) [16]. In addition to increasing food intake, sleep restriction is responsible for causing endocrine and metabolic alterations, including decreased glucose tolerance, decreased insulin sensitivity [21,22], and increased evening concentrations of cortisol [23,24]. In addition, epidemiologic data correlate short sleep habits with the onset of metabolic-related disorders such as obesity and type 2 diabetes Mellitus, T2DM [25,26]. It has also been suggested that sleep restriction may be an important regulator of energy metabolism in peripheral tissues by increasing insulin resistance in human adipocytes [27]. However, to date, very little is known about the effect of sleep loss on WAT metabolic homeostasis, especially at the molecular level. Given the progressive decrease in sleep time and the concomitant marked increase in obesity witnessed in the last decades in modern societies [15,16,17], investigating the causal link and the physiological mechanisms involved in sleep loss and WAT metabolism could lead to the identification of new strategies to ameliorate health. Here, we investigated whether chronic sleep restriction (CSR), a condition of prolonged short sleep, affects the transcriptional regulation of SIRT1 signaling in mice WAT. Gene expression analysis was used to identify key SIRT1-regulated metabolic pathways in WAT. The data demonstrated that CSR increased the expression levels of SIRT1 and some of its transcriptional targets (i.e., PPARs, PGC-1A, and sterol regulatory element-binding transcription factor 1/2-SREBF) involved in the lipid metabolism and mitochondrial functions of adipocytes. These data provide new insights into how SIRT1 signaling regulates adipogenic pathways in sleep-restricted mice.

## 2. Materials and Methods

### 2.1. Animals

Male C57BL/6J mice (14 weeks old) were used for the experiment. Mice were housed in groups of two in environmentally controlled cages for the duration of the experiment. Environmental conditions were as follows: 12 h light/dark cycle, light on at 8:00 p.m.; temperature of 24 ± 1 °C; food (standard laboratory chow, VWR International, LLC, Radnor, PA, USA, Cat N. 470105-576) and water available ad libitum and replaced daily at 9:00 a.m. The body weights of mice were measured before and after experimental conditions. The food intake of mice was measured daily by recording the amount of chow consumed per cage. All the experiments were performed according to the local Institutional Animal care and Use Committee and the European Communities Council Directives (2010/63/EU, protocol P5E96A446) and complied with the 3Rs Australian Code for the care and use of animals for scientific purposes (Replacement of animals with alternatives wherever possible, Reduction in number of animals used, and Refinement of experimental conditions and procedures to minimize the harm to animals) [28].

### 2.2. Experimental Conditions

**Figure 1:**
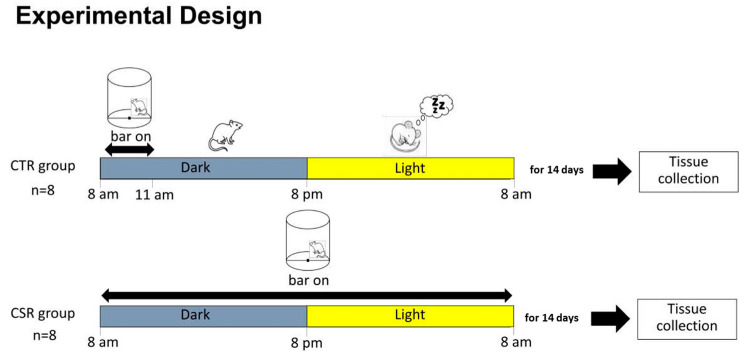
Experimental design. Young adult mice were divided into two groups: Chronic sleep restriction (CSR) and Control group (CTR). Mice were exposed to either CSR or 3 h bar movement for 14 days, then tissue was collected for molecular analysis.

The experimental design is summarized in Figure 1. Mice were weight-balanced and divided into two experimental groups: (1) chronic sleep-restricted (CSR) group (n = 8), with a mean body weight of 23.9 g (95% CI: 23.1, 24.8). In this group, mice were sleep-restricted for 14 days (24/24 h) using an automated sleep deprivation chamber (Pinnacle Technology Inc., Lawrence, KS, USA). The effectiveness of this automated sleep deprivation method has been proved in previous experiments using EEG recording in rodents [29,30]. The procedure consists of a rotating bar placed at a short distance above the cage floor, lightly nudging the animal from sleep and encouraging low levels of activity until the animal maintains wakefulness on its own. The rotating bar was set counterclockwise, at a velocity of 2 rpm (one turn each 30 s). (2) Control (CTR) group (n = 8), with a mean body weight of 22.9 g (95% CI: 21.9, 24.0). In this group, mice were placed in the same sleep deprivation chambers and allowed to sleep undisturbed, except for 3 h/day (during the dark period, when mice are usually awake), during which the bar rotation was activated to expose the mice of this group to the experience of the bar movement and the stress associated with it. Animal behavior was assessed daily by two different operators through direct visual observation, aiming to minimize subjective bias. After 14 days, all mice were sacrificed between 9:00 and 11:00 a.m. to maintain the time of tissue collection within the same 2 h time window for all experimental groups.

### 2.3. WAT Collection

All mice were deeply anesthetized with isoflurane 3%. Mice were sacrificed by cervical dislocation, immediately immersed in a freezing 2-methylbutane solution for a few seconds and dissected for WAT collection. To access visceral organs, the central skin near the genital organs was elevated and a small incision was performed. Then, the scissor was put horizontally into the opened hole, allowing the cutting of the abdominal muscle along the linea alba (for ~4 cm, depending on the mouse size) from the genitals to the rib cage. Consistently, WAT was collected in the same specific area for all mice. The fat used in this study was perigonadal fat (PGF), localized around the reproductive organs. PGF was collected by separating it from surrounding structures (epididymis, the testis, and the ductus deferens). All samples were immediately frozen in dry ice and subsequently stored in a −80 °C freezer for later analysis.

### 2.4. Molecular Analyses 

Total RNA was extracted from 50 mg of WAT using 0.5 mL QIAzol^®^ Lysis Reagent (Qiagen, Germantown, MD, USA) according to the manufacturer’s instructions (QIAzol^®^ Lysis Reagent, QIAGEN^®^ Sample & Assay Technologies). RNA concentration and purity were assessed spectrophotometrically at an absorbance of 260/280 nm, and the integrity was confirmed by electrophoresis through 1% agarose gels stained with ethidium bromide. The complementary DNA (cDNA) was synthesized from 2 µg of total RNA in 20 µL of total volume reaction using 4 µL of 5X All-In-One RT MasterMix (abm, Richmond, BC, Canada) according to the manufacturer’s instructions (abm^®^, Cat. No. G592). The optimized 5X RT MasterMix contains abm’s proprietary OneScript^®^ Hot Reverse Transcriptase, RNaseOFF Ribonuclease Inhibitor, temperature-sensitive DNase, dNTPs, and a finely balanced ratio of Oligo (dT)s and Random Primers. The mixture was incubated at 37 °C for 15 min, followed by 45 °C for 60 min. The cDNA synthesis reaction was stopped by heating at 85 °C for 2 min. SYBR green-based real-time PCR (q-PCR) was used to evaluate expression profiles of PPARG, PPARA, SIRT-1, LEPT, PGC-1A, PRDM16, C-Myc, SREBF, CEBP, UCP-1, and ADIPONECTIN target genes. The most stable housekeeping gene, Glucose-6-phosphate dehydrogenase (G6PDH), was used as a reference gene for the qPCR analysis [31]. All the primer sequences are reported in Table 1. The expression of individual gene targets was analyzed using the ABI 7300 Real-Time PCR System (Applied Biosystems Inc., Waltham, MA, USA). All reactions were prepared in 20 µL of total volume using BlasTaq™ 2X qPCR MasterMix according to the manufacturer’s instructions (abm, Richmond, BC, Canada). Briefly, the reaction included 10 μL BlasTaq™ 2X qPCR MM (Abm^®^, Richmond, BC, Canada), 10 μmol L^−1^ each of forward and reverse primers, 1 μL cDNA template, and sterile distilled water (abm^®^, Richmond, BC, Canada). Thermocycling for all reactions was for 3 min at 95 °C, followed by 40 cycles of 15 s at 95 °C and of 60 s at 60 °C. Fluorescence was monitored at the end of every cycle. Melting curve analysis was performed to confirm the presence of a single amplicon of interest. Results were calculated using the relative 2^−ΔΔCt^ method [32] and means of mRNA levels were expressed with respect to control mice ± standard deviation (SD).

### 2.5. STRING Network Visualization

A cluster analysis of the genes selected in the present study was performed using the K-means clustering method in STRING (Search Tool for the Retrieval of Interacting Genes/Proteins) [40,41], only including interactions with a confidence score higher than 0.7. The STRING v11.5 web resource [42] is a database which gives an association score for interacting proteins, which is calculated using various parameters such as neighborhood score, fusion, co-occurrence, homology, co-expression, experimental, database, and text mining score [43]. Recently, STRING v11.5 has been also successfully applied to identify gene interaction networks [41,44]. To simplify the dense network and to obtain a better visual representation and comprehension of the interactions, the resulting groups were manually separated.

### 2.6. Statistical Analysis 

q-PCR results were expressed as the normalized fold change corrected for G6PDH and with respect to the control group (2^−ΔΔCt^ method). Data were first examined for their fit to a normal distribution and homogeneity of variance using Kolmogorov–Smirnov and Levene median tests. Student’s *t*-test was used to determine statistical differences between CSR and Control group. All statistical analyses were performed with GraphPad Prism 8.0 (GraphPad Software, Inc., San Diego, CA, USA).

## 3. Results

**Figure 2:**
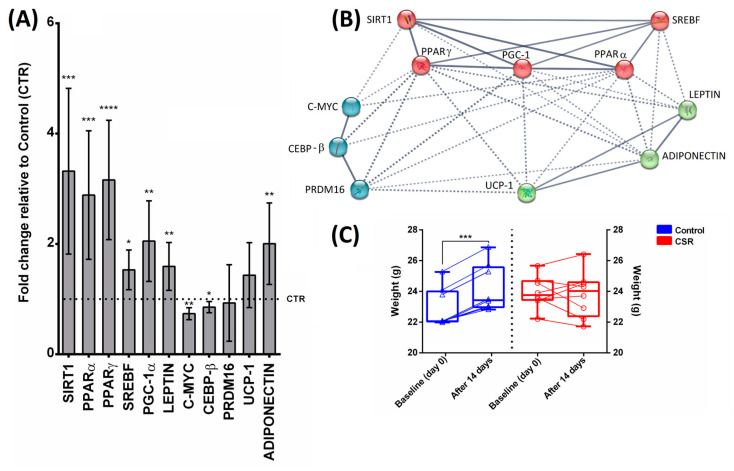
Effects of chronic sleep restriction (CSR) on mRNA expression of white adipose tissue (WAT)-related genes (**A**) and body weights (**C**). Sirtuin-1 (SIRT1); peroxisome proliferator-activated receptor gamma (PPARγ); peroxisome proliferator-activated receptor alpha (PPARα); sterol regulatory element-binding transcription factor 1/2 (SREBF); peroxisome proliferator-activated receptor gamma coactivator 1-alpha (PGC-1α); leptin (LEPT); CCAAT/enhancer-binding protein beta (CEBP-β); Myc (C-Myc); PR domain containing 16 (PRDM16); Uncoupling Protein 1 (UCP1); adiponectin. Gene expression is shown as fold changes relative to the control group. Data are means ± 95% C.I. (n = 8). * *p* < 0.05, ** *p* < 0.01, *** *p* < 0.001, **** *p* < 0.0001. Clustered gene association network using STRING v11.5 (**B**). The solid and dotted lines indicate connections within the same and different clusters, respectively.

The expression levels of SIRT1 were significantly increased in CSR mice with respect to the control animals (Figure 2A, *p* < 0.001). Similarly, the signaling pathway downstream from activated SIRT1 (i.e., PPARα, PPARγ, PGC-1α, LEPT, ADIPONECTIN, and SREBF) was significantly up-regulated by sleep restriction (Figure 2A, *p* < 0.05). On the contrary, CEBP-β and C-myc were down-regulated in sleep-restricted mice (Figure 2A, *p* < 0.05). Both PRDM16 and UCP-1 did not show any significant difference in mRNA levels between the experimental groups (Figure 2A). The K-means clustering analysis in STRING identified four main clusters (Figure 2B). All genes within and between each cluster are strongly interconnected, reflecting a high degree of functional association, and suggesting an interplay among the numerous pathways related to the gene network. In this regard, we identified genes that (1) are involved in the transcriptional regulation of lipid homeostasis (red group), (2) are defined as DNA-binding transcription factors during the adipogenesis process (light blue group), and (3) act as regulators for adipose tissue development (green group) (Figure 2B). Our results also showed that the body weights of CSR mice remained almost unchanged after 14 days of treatment, while the body weights of control mice were found to be significantly increased at the end of the experiment (Figure 2C, *p* < 0.001). Regarding food consumption, no significant differences were found between groups, with a mean food intake of 3.5 ± 0.3 g/day in CTR mice and 4.2 ± 1.1 g/day in CSR mice.

## 4. Discussion

In this study, we investigated the effects of CSR on SIRT1-dependent molecular pathways in the WAT of mice models. Our findings are consistent with the role of SIRT1 activation in adipose tissue signaling as previously suggested by different studies and well described by Boutant and Cantó [7]. Indeed, the up-regulation of SIRT1 was found to decrease the mRNA levels of key WAT transcription factors and up-regulate those of brown adipose tissue (BAT) genes. Importantly, the genes whose expression is induced by SIRT1 activation include those involved in mitochondrial biogenesis, such as PGC-1α, and fatty acid oxidation, such as PPARα [9,45]. Majeed et al. [10] reported that PGC-1α expression levels were promoted by SIRT1, as demonstrated using SIRT1-depleted adipocyte models which, in turn, showed a reduced mitochondrial mass and respiratory capacity. On the other hand, the activation of PPARα is pivotal in regulating lipid metabolism and promoting fatty acid oxidation [45]. The SIRT1:PPARα interaction was protective in cardiac hypertrophy [46] and the deregulation of its related signaling pathway was associated with reduced fatty acid oxidation in the liver [47]. Interestingly, the CSR-induced up-regulation of PPARα paired with that of SREBF. As previously demonstrated, SREBF1 overexpression in 3T3-L1 adipocytes promoted fatty acid metabolism and resulted in the production of lipids that work as PPARγ ligands [48,49]. Therefore, these findings could explain, at least in part, the increase in PPARγ expression observed in our study. There is, indeed, evidence that, in WAT, SIRT1 usually acts as a PPARγ repressor rather than an inducer [50]. This effect is most likely due to a SIRT1-dependent deacetylation of PPARγ that subsequently causes the recruitment of PRDM16, a transcriptional co-regulator of the BAT genetic program. Importantly, the SIRT1-dependent deacetylation of PPARγ is responsible for UCP-1 induction in WAT [51]. However, we found no change in UCP-1 nor in PDRM16 expression, which could indicate unaffected acetylation levels of PPARγ in WAT. Other studies found increased UCP mRNA and protein levels in the BAT and muscles of chronic sleep-restricted rats, suggesting the increased energy expenditure associated with prolonged sleep loss [52,53].

Our study also indicates that sleep restriction significantly increased the expression of the leptin and adiponectin adipokines in the CSR group with respect to the control mice. From a molecular point of view, it is possible that a SIRT1-mediated increase in CEBP-α transcriptional activity is responsible for the induction of the leptin gene in response to CSR. The stimulation of leptin transcription was previously found to be SIRT1-dependent in preadipocytes supplemented with the NAD+ booster, nicotinamide mononucleotide (NMN) [10]. In addition, leptin expression is highly correlated with PPARγ expression, which was also increased in CSR mice [54]. However, in the literature, there are conflicting results regarding leptin circulation in sleep curtailment, with its levels found to be both reduced [55,56,57] and elevated [58,59,60,61]. These discrepancies may be related to the duration and quality of sleep disturbances applied. Qiao and Shao [62] have demonstrated that SIRT1 can also be involved in up-regulating adiponectin expression by inducing Foxo1 transactivation activity. We can thus speculate that, with CSR, SIRT1 expression is induced, which leads to the mobilization of fatty acids in WAT and the increased transcription of adiponectin. Adiponectin, in turn, enhances insulin sensitivity, further improving fatty acid oxidation [63].

Overall, our data suggest the presence of functional interactions among SIRT1, PPARα, SREBF, and PGC-1α that result in reduced fat accumulation and enhanced fat consumption, probably due to the stimulation of the adipose thermogenic capacity. Together with leptin, all these genes act as metabolic regulators whose expression patterns are sensitive to CSR in a SIRT1-dependent manner. The CSR-induced downregulation of genes associated with the adipogenic pathway (i.e., CEBP-β and C-Myc) is also consistent with the reduction in fat accumulation. Earlier evidence has shown that adipogenesis, as well as adipocyte differentiation, are driven by two subsequent waves of transcription factor activation during which CEBP-β has a pivotal function [64,65]. CEBP-β is a well-known early mediator of adipocyte differentiation and is involved in the activation of several targets, including PPARγ, which, in turn, mediate adipogenic differentiation [66]. Interestingly, CEBP-β shows polylysine acetylation, which may be a deacetylation target of SIRT1 [67]. Consequently, SIRT1-dependent deacetylation alters CEBP-β function as a transcription factor in adipocytes. In line with this, SIRT1 was shown to inhibit C-Myc signaling, as demonstrated using SIRT1-silenced preadipocytes [68]. The SIRT1-mediated deacetylation of C-Myc resulted in reduced preadipocyte hyperplasia, lipid accumulation, and inflammation [68]. In addition, Tóth et al. [69] suggested that C-Myc could participate in the regulation of the adipocyte–thermogenic function by modulating UCP1 gene expression. Overall, the CSR-induced overexpression of SIRT1 matches the repression of both CEBP-β and C-Myc.

Chronic sleep restriction in rodents is known to cause a cluster of syndromes first described by Rechtschaffen and Bergmann [70] and that include hyperphagia, weight loss, elevated energy expenditure, increased plasma catecholamines, hypothyroidism, reduction in core temperature, deterioration in physical appearance [71], reduced levels of anabolic hormones [72], and declines in the integrity of the immune system [73]. Reduced weight gain occurs consistently, despite unchanged or increased levels of food consumption [52,74,75]. Accordingly, our results suggest that the induction of SIRT1-dependent molecular pathways prevents weight gain. Indeed, in the present work, CSR causes trouble in gaining weight, despite food intake remaining unchanged. Our findings also suggest that, in addition to the increased energy expenditure associated with sleep restriction, the overexpression of SIRT1 signaling could be involved in regulating lipid metabolism, mostly by inducing a decrease in fat accumulation leading to a new energy homeostasis. As a whole, the obtained gene network highlights the crucial role of SIRT1-dependent molecular pathways in mediating the metabolic remodeling of adipose tissue induced by CSR.

## 5. Conclusions

Altogether, our results indicate that CSR induces the SIRT1-mediated metabolic remodeling of WAT. The overexpression of SIRT1 decreases lipid accumulation, probably by inducing fat oxidation and increasing adaptive thermogenesis. However, further studies are needed to characterize the temporal dynamics of thermogenic gene expression and the long-term effects of WAT adaptation to CSR.

## Figures and Tables

**Table 1:** Primer sequences (5′-3′) used for qPCR analysis.

Gene	Primer Sequence (5′-3′)	GenBank	Reference
PPARγ	ATGGAGCCTAAGTTTGAGTTTGCT GGATGTCCTCGATGGGCTTCA	XM_006505737.5	[33]
PPARα	CACCCTCTCTCCAGCTTCCA GCCTTGTCCCCACATATTCG	XM_011245516.4	[34]
SIRT-1	CGATGACAGAACGTCACACG TCGAGGATCGGTGCCAATCA	NM_019812.3	[35]
LEPT	GACATTTCACACAGGCAGTCG GCAAGCTGGTGAGGATCTGT	NM_008493.3	[31]
ADIPONECTIN	GAGAAGGGAGACGCAGGTGT GCTGAATGCTGAGTGATACATGTAAG	XM_032899235.1	[31]
SREBF	GAACAGACACTGGCCGAGAT GAGGCCAGAGAAGCAGAAGAG	NM_011480.4	[36]
CEBP-β	ACCGGGTTTCGGGACTTGA GTTGCGTAGTCCCGTGTCCA	X62600.1	[37]
C-Myc	GCCACGTCTCCACACATCAG TGGTGCATTTTCGGTTGTTG	XM_021216459.1	[38]
PRDM16	CACGGTGAAGCCATTCATATGCG AGGTTGGAGAACTGCGTGTAGG	XM_006539171.5	[35]
UCP-1	GCCATCTGCATGGGATCAAACC TCGTCCCTTTCCAAAGTGTTGAC	NM_009463.3	[35]
PGC-1α	CTCCAGCCTGACGGCACCC GCAGGGACGTCTTTGTGGCT	NM_001330751.2	[39]
G6PDH	ATTGACCACTACCTGGGCAA GAGATACACTTCAACACTTTGACCT	XM_021153829.2	[31]

## Data Availability

The data presented in this study are available on request from the corresponding author.
